# Neonatal inflammation increases hippocampal KCC2 expression through methylation-mediated TGF-β1 downregulation leading to impaired hippocampal cognitive function and synaptic plasticity in adult mice

**DOI:** 10.1186/s12974-023-02697-x

**Published:** 2023-01-23

**Authors:** Jing Rong, Yang Yang, Min Liang, Haiquan Zhong, Yingchun Li, Yichao Zhu, Sha Sha, Lei Chen, Rong Zhou

**Affiliations:** Department of Physiology, Nanjing Medical University, Longmian Avenue 101, Jiangning District, Nanjing, 211166 Jiangsu China

**Keywords:** Neonatal inflammation, Memory, GABAergic synaptic transmission, Cytokines, Methylation

## Abstract

The mechanisms by which neonatal inflammation leads to cognitive deficits in adulthood remain poorly understood. Inhibitory GABAergic synaptic transmission plays a vital role in controlling learning, memory and synaptic plasticity. Since early-life inflammation has been reported to adversely affect the GABAergic synaptic transmission, the aim of this study was to investigate whether and how neonatal inflammation affects GABAergic synaptic transmission resulting in cognitive impairment. Neonatal mice received a daily subcutaneous injection of lipopolysaccharide (LPS, 50 μg/kg) or saline on postnatal days 3–5. It was found that blocking GABAergic synaptic transmission reversed the deficit in hippocampus-dependent memory or the induction failure of long-term potentiation in the dorsal CA1 in adult LPS mice. An increase of mIPSCs amplitude was further detected in adult LPS mice indicative of postsynaptic potentiation of GABAergic transmission. Additionally, neonatal LPS resulted in the increased expression and function of K^+^–Cl^−^-cotransporter 2 (KCC2) and the decreased expression of transforming growth factor-beta 1 (TGF-β1) in the dorsal CA1 during adulthood. The local TGF-β1 overexpression improved KCC2 expression and function, synaptic plasticity and memory of adult LPS mice. Adult LPS mice show hypermethylation of *TGFb1* promoter and negatively correlate with reduced TGF-β1 transcripts. 5-Aza-deoxycytidine restored the changes in *TGFb1* promoter methylation and TGF-β1 expression. Altogether, the results suggest that hypermethylation-induced reduction of TGF-β1 leads to enhanced GABAergic synaptic inhibition through increased KCC2 expression, which is a underlying mechanism of neonatal inflammation-induced hippocampus-dependent memory impairment in adult mice.

**Supplementary Information:**

The online version contains supplementary material available at 10.1186/s12974-023-02697-x.

## Background

During the early stages of brain development including prenatal and neonatal periods, infection/inflammation may have significant consequences for the development and function of physiological systems throughout an individual’s lifespan, a phenomenon termed “perinatal programming” [1, 2]. For instance, neonatal exposure to bacterial products (lipopolysaccharide, LPS) in rats influences reactivity to stress and immune regulation [3]. Furthermore, an increasing body of evidence from both animal and human studies suggests that perinatal inflammation may contribute to the development of schizophrenia [4, 5], autism [6, 7], bipolar disorder [8] and cognitive deficits [2, 9, 10]. To date, the mechanisms by which perinatal inflammation induces neuropsychiatric disorders have been widely concerned. However, few studies have focused on the mechanisms of cognitive impairment due to inflammation that occurs in perinatal period, especially in neonatal period.

Several studies have shown that the various alterations of inhibitory synaptic transmission mediated by γ-aminobutyric acid (GABA) in prefrontal cortex, forebrain, and hippocampus are key links between perinatal inflammation and neuropsychiatric disorders [11–14]. This also suggests that GABAergic synaptic transmission is one of the neuronal networks that is highly sensitive to perinatal inflammation. It is well known that GABA neurotransmission is involved in the regulation of emotion, movement initiation, and memory via controlling neuronal excitability, integration, and plasticity [15–17]. GABA type A receptor (GABA_A_R) is the main receptor that mediates GABAergic signaling. The dynamic regulation of the intracellular chloride concentration by Na^+^–K^+^–Cl^−^ cotransporter 1 (NKCC1) and K^+^–Cl^−^ cotransporter 2 (KCC2), which determines the polarity and efficacy of GABAergic inhibition. Multiple lines of evidence suggest the deficits in GABA_A_R-mediated transmission are involved in the etiology of various neurological disorders such as autism, schizophrenia, epilepsy and Parkinson’s disease [18–22]. It is important to note that the alterations in GABAergic functions are most likely not limited to these disorders. For example, preclinical studies have found that there is a significant increase of GABA release in mouse models of neurofibromatosis Type 1 and Down syndrome, two neurodevelopmental disorders associated with cognitive impairments [23, 24]. Pharmacological findings have showed that positive allosteric modulators of GABA_A_R impair memory processing [25–27], whereas GABA_A_R blockers or inverse agonists often potentiate cognitive and memory performance [28, 29]. These findings suggest the close relation between the GABA_A_R-mediated over-inhibition and cognitive impairments. Based on the analysis above, it is speculated that perinatal inflammation-related cognitive deficits are likely to be affected by GABAergic synaptic dysregulation.

Additionally, evidence exists in support of the role of proinflammatory cytokines in social, cognitive and emotional behavioral deficits [7, 30–32]. For example, interleukin-1beta (IL-1β), IL-6 and tumor necrosis factor alpha (TNF-α) disrupt brain structures connectivity involved in social, emotional and memory processes [33–35]. Conversely, transforming growth factor-beta 1 (TGF-β1), an anti-inflammatory cytokine is a powerful regulator of learning, memory, and synaptic plasticity [36–38]. It has been found that postnatal inflammation induces the alterations of anti/pro-inflammatory cytokine expression in the rat pup and adult brain [39].

It is well known that hippocampus is divided into dorsal hippocampus and ventral hippocampus. CA1 region of dorsal hippocampus is fundamental for contextual and spatial cognition, whereas CA1 of ventral hippocampus plays a role in processing emotional and motivational behaviors [40–42]. Consequently, the current study focused on dorsal CA1 region to investigate the link among pro/anti-inflammation cytokines, GABAergic synaptic transmission and adult cognitive function to determine the molecular mechanisms underlying neonatal inflammation-induced cognitive deficits.

## Materials and methods

The present studies were approved by Animal Care and Use Committee of Nanjing Medical University. The protocols used here were in accordance with the guidelines published in the NIH Guide for the Care and Use of Laboratory Animals. All efforts were made to minimize the number of animals and their suffering.

### Animal model preparation

Following a 2-week period of acclimatization to the new animal housing room, to facilitate the mating, male and female C57BL/6 mice (Animal Core Facility, China) were kept together one-by-one in a cage. Pregnancy in female mice was determined by visual detection of the vaginal plug. When pregnancy was confirmed, the female mice were removed from the breeding cages and housed individually in standard cages. All pregnant animals were allowed to have normal delivery and the first day of birth was considered postnatal day (P) 0. Male pups received a daily subcutaneous injection of 50 μg/kg lipopolysaccharide (LPS, dissolved in 0.9% saline, Sigma-Aldrich) from P3 to P5 (neonatal period). 1 ml/kg saline was used as the vehicle control. Both dose and time of LPS treatment in newborn mice were chosen based on the previous studies [43, 44]. Neonate mice were returned to their housing immediately following saline (control mice) or LPS administration (LPS mice). The body weights of the mice were regularly monitored, and no obvious difference in the body weight was observed at different developmental stages (P3, 10, 28, 45 and 70) between control and LPS mice. Only one male mouse per dam was used for each of the experiments to avoid the litter-effect. Since cognitive impairments caused by perinatal inflammation occur in adulthood [9, 10], almost all experiments in the present study were carried out in adulthood (P70–P87). In addition, it was determined at P8 whether the systemic administration of LPS during neonatal period caused acute inflammation of dorsal CA1. In the present study, the schematic diagram of the experimental design and timeline for each section was showed together with the corresponding results.

### Behavior analysis

#### Contextual/cued fear conditioning

Animals were placed in the fear conditioning apparatus (Panlab) for 2 min. Then, a 30-s acoustic conditioned stimulus (CS; 80 dB tone) was delivered, and a 0.5-mA shock unconditioned stimulus (US) was applied to the floor grid during the last 2 s of the CS. Training consisted of two CS–US pairings, with a 1.5-min interval between each. On day 2, mice were placed back into the original conditioning chamber for 5 min (no shocks or conditioned stimulus given). Immediately after the contextual test, mice were placed into a novel context and exposed to the CS for 3 min. The time spent on freezing behavior (i.e., remain motionless for at least 2 s) was recorded in the original conditioning chamber or during presentation of the CS.

#### Morris water maze

The Morris hidden-platform water maze consisted of a circular pool (1.38 m diameter) filled with nontoxic opaque water at room temperature with an escape platform (10 cm diameter) hidden beneath the water (3 cm). Each mouse was placed in the pool in a pseudorandom order and given 60 s to locate the escape platform. When the mouse found the platform or if the mouse failed to find the platform within 60 s, it was placed on the platform where it remained for 30 s. The mouse was then dried and returned to the cage to rest for 15 min before the next trial. Each mouse was given four trials per day, with an intertrial interval of 1 h. The time to find the platform (escape latency), the total distance traveled, and the swim speed of the animals were recorded. The mice were then towel dried and placed in a cage with a heating pad underneath until dry and returned to their home cage. On day 8, all mice were subjected to one probe trial in which the platform was removed, and each animal had 60 s to search the pool for the platform.

### Histological staining

#### Slice preparation

Mice were deeply anaesthetized with pentobarbital (50 mg/kg, i.p.) and perfused transcardially with 4% paraformaldehyde. Some brains were post-fixed in 4% paraformaldehyde for 24 h, after which they were transferred into 30% sucrose in PBS for at least 48 h at 4 °C. Exhaustive coronal sections (40-μm-thick) containing dorsal hippocampus were made using a Microm cryostat (Richard-Allan Scientific) and stored in cryoprotectant at 4 °C. Other brains were processed for paraffin embedding. Coronal sections (5-μm-thick) containing hippocampus were cut.

#### Toluidine blue staining

The 40-μm-thick sections were washed in PBS and stained with toluidine blue. Area of the hippocampus was measured with using image analysis software (NIH-Image 3.12) and multiplied by the thickness of section to calculate the volume of each section. The volume of the dorsal hippocampus was obtained by adding the volumes of all sections. The 5-μm-thick sections were stained with toluidine blue to examine the structural changes of the dorsal hippocampus.

#### Immunohistochemical labeling

For blocking and permeabilization in 40-μm-thick sections, we used “staining buffer” containing 0.05 M Tris, 0.9% NaCl, 0.25% gelatin, and 0.5% Triton X-100, pH 7.4. Primary antibodies, rabbit anti-KCC2 (1:500; Sigma-Aldrich) were diluted in staining buffer and incubated overnight at 4 °C. The next day, the sections were washed in PBS and incubated with donkey anti-rabbit FITC antibody (1:500; Sigma-Aldrich). After a final three washes, sections were mounted in antifade mounting medium. Immunofluorescence-labeled cells were observed using a confocal laser-scanning microscope (Leica). KCC2 staining intensities in the CA1 region were measured using NIH Image J software. A box was drawn in the pyramidal cell body layer of CA1 field was quantified. Three independent background fluorescence intensity measurements were averaged and subtracted from the fluorescence intensity of each cell. KCC2 expression was estimated by calculating the relative mean fluorescence intensities per unit area.

### Biochemical detection

#### Tissue preparation

The brain was rapidly removed after decapitation and placed on glass petri dish filled with ice-cold saline, then hippocampus tissues were isolated. Finally, CA1 area of dorsal hippocampus was carefully dissected and rapidly frozen in liquid nitrogen before storage at − 80 °C.

#### Real-time RT-PCR

Total RNA was extracted using TRIzol (Invitrogen) following the manufacturer’s instructions. Possible contamination with genomic DNA was removed by an on-column DNase I (Qiagen) treatment. mRNA was reverse transcribed using the high-capacity cDNA Reverse Transcription kit (Applied Biosystems) following the manufacturer’s instructions. The primer sequences are as follows: *TGFb1* (TGF-β1): forward, CCCTATATTTGGAGCCTGGA, reverse, CTTGCGACCCACGTAGTAGA. *Tnf* (TNF-α): forward, ACAGAAAGCATGATCCGCG, reverse, GCCCCCCATCTTTTGGG. *Il1b* (IL-1β), forward, GCACACCCACCCTGCAG, reverse, AACCGCTTTTCCATCTTCTTCTT. *Gapdh* (GAPDH): forward, AGGTCGGTGTGAACGGATTTG, reverse, GGGGTCGTTGATGGCAACA. RT-PCR was performed using a LightCycler FastStart DNA Master SYBR Green I kit (Roche) and an ABI Prism 7300 Sequence Detection System (Applied Biosystems). To improve the accuracy of the real-time PCR for quantification, amplifications were performed in triplicate for each RNA sample. mRNA levels was determined using the 2-ΔΔCt method with normalization to *Gapdh*.

#### Western blot

The protein was extracted using a total protein extraction kit (KeyGEN Biotech), and protein concentration was determined with the BCA Protein Assay Kit (Pierce Biotechnology). Total protein (20–50 μg) was separated by SDS–polyacrylamide gel electrophoresis and transferred to a polyvinylidene difluoride membrane. The membranes were blocked with 5% nonfat dried milk for 60 min and then incubated in the antibodies of rabbit anti-GABA_A_Rα1 (1:1000, Abcam), rabbit anti-GABA_A_Rα2 (1:1000, Abcam), rabbit anti-NKCC1 (1:400, Cell Signaling Technology), rabbit anti-KCC2 (1:8000, Abcam), rabbit anti-IL-1β (1:1000, Abcam), rabbit anti-TNF-α (1:1000, Abcam), mouse anti-TGF-β1 (1:200, Affinity) and mouse anti-β-actin antibody (1:5000, Abways Technology) as loading control. After thorough washing, the membranes were incubated with HRP-labeled secondary antibodies and developed using the ECL detection Kit (Millipore). Western blotting bands were scanned and analyzed using the Image J analysis software package (NIH). The densitometric values of the total protein were normalized by β-actin.

#### Methylated/hydroxymethylated DNA immunoprecipitation

Methylated (5-mC) and hydroxymethylated DNA (5-hmC) on *TGFb1* promoter were assessed using MeDIP (Diagenode) and hMeDIP kits (Diagenode) followed by quantitative PCR. Briefly, 10 μg of ChIP grade 5-mC antibody or 5-hmC antibody was added to the sonicated solution and incubation at RT for 60 min. An aliquot of the sonicated lysate without antibody was used as input to quantify the total amount of DNA in sample extracts. Protein-free DNA was extracted and used for detection and quantification of 5-mC/5-hmC at *TGFb1* promoter by quantitative PCR. The primers of CpG-rich *TGFb1* promoter were as follows: forward primer, 5′- GCCCACGCTAAGATGAAGAC -3′; reverse primer, 5′- CTCCTCGGCTGCTCCTTT -3′. The 5-mC/5-hmC level at *TGFb1* promoter was expressed as a percentage of the input DNA using the following equation: % (DNA-IP/total input) = 2 ^[(Ct(10%input) − 3.32) − Ct (DNA−IP)]^ × 100%.

### Electrophysiological analysis

#### Slice preparation

Mice were killed by decapitation, and prepared for slices as described previously [45]. Their brains were quickly removed and placed in ice-cold oxygenated artificial cerebrospinal fluid (ACSF) consisting of (in mM) 124 NaCl, 2 CaCl_2_, 4.5 KCl, 1.0 MgCl_2_, 26 NaHCO_3_, 1.2 NaH_2_PO_4_, and 10 D-glucose and adjusted to pH 7.4 by bubbling with 95% O_2_/5% CO_2_ mixture. Coronal brain slices (400-μm-thick) were cut using a vibrating microtome in ice-cold oxygenated (95% O_2_/5% CO_2_) ACSF. Slices from dorsal hippocampus were stored for a minimum of 1 h in oxygenated ACSF maintained at 30 ± 1 °C prior to extracellular recording or whole-cell patch-clamp recording.

#### Extracellular recording

The hippocampal slices were transferred to a chamber continuously perfused with oxygenated ACSF (2 ml/min) maintained at 30 °C. Field excitatory postsynaptic potentials (fEPSPs) were obtained from area CA1 stratum radiatum with the use of a glass microelectrode filled with ACSF (4–5 MΩ). fEPSPs were evoked through stimulation of the Schaffer collaterals using a 0.1 ms biphasic pulse delivered every 20 s. After a consistent response to a current stimulus was established, threshold current for evoking fEPSPs was determined, and the current was increased incrementally every 0.1 mA until the maximum amplitude of the fEPSPs was reached (input/output curve). All other stimulation paradigms were induced at the same current intensity, defined as 50% of the stimulus current used to produce the maximum fEPSPs amplitude, for each individual slice. Paired-pulse facilitation (PPF) was induced by two paired pulses with the interval at 20, 50, 75, 100 ms. A fEPSPs long-term potentiation (LTP) baseline response was recorded every 15 s for 20 min. The tetanus used to evoke LTP was high-frequency stimulation (HFS), which consisted of two trains of 1 s, 100 Hz pulses, with an intertrain interval of 20 s. Following HFS, fEPSPs were recorded for 60 min. The fEPSPs descending slopes were averaged into 1-min bins and graphed. The slopes of fEPSPs were normalized to the mean fEPSPs descending slope of baseline recordings. Successful induction LTP required the fEPSPs slopes at 60 min post-HFS greater than 120%.

#### Whole-cell patch-clamp recording

The hippocampal slices were transferred to a chamber continuously perfused with oxygenated ACSF (2 ml/min) maintained at 30 °C. Recordings were obtained from CA1 pyramidal neurons. Signals were amplified using an EPC-10 amplifier (HEKA Elektronik) and analyzed using PulseFit software (HEKA Elektronik). Electrodes (5–8 MΩ resistance) were filled with a solution containing the following (in mM): 130 K^+^-gluconate, 10 KCl, 10 HEPES, 0.2 EGTA, 4 ATP, 0.3 GTP and 10 phosphocreatine, pH 7.4. Pipettes were dipped into this solution for several seconds and then backfilled with the same solution containing gramicidin (10 μg/ml, Sigma-Aldrich). A GABA-activated current (*I*_GABA_) induced by puffing GABA (10 μM) at the cell body of principal neurons with holding potential at − 80 mV. To determine the reversal potential for *I*_GABA_ (*E*_GABA_), the membrane potential was stepped in + 20 mV increments from − 120 to − 20 mV and the amplitude of *I*_GABA_ in response to exogenous GABA puffs was measured. Miniature inhibitory postsynaptic currents (mIPSCs) were recorded with holding potential at resting potential level. For resting membrane potential, the amplifier was set to *I* = 0 and the corresponding potential was measured under current clamp mode. mIPSCs were analyzed by Mini Analysis software (Synaptosoft Inc.). mIPSC frequency and amplitude were sampled for periods of 100 s. Rise time and decay time kinetics were analyzed using average mIPSCs waveform of 100 randomly selected synaptic events per neuron. Rise time was defined as the duration of the rise from 10 to 90% of the peak of average mIPSCs waveform. Decay time was defined as the time for average mIPSCs to decay to half-amplitude.

### Drug administration

GABA_A_R antagonist picrotoxin (PTX) and methylation inhibitor 5-aza-deoxycytidine (5-aza-CdR) were purchased from Sigma-Aldrich. CMV-Tgfb1-eGFP and CMV-eGFP plasmids were packaged into adenoviral (ADV) capsids by GeneChem of China. For the bath-application of the slices, PTX (10 µM) were dissolved to their final concentration in ACSF. Drug solutions entered the recording chamber through the perfusion tubing. In vivo administration, PTX (5 mg/kg) or vehicle was intraperitoneally (i.p.) injected. For micro-injection, a guide cannula (26-gauge, Plastics One) was implanted into the CA1 field of dorsal hippocampus (bregma, − 1.9 mm; 1.4 mm lateral, ± 1.1 mm lateral; ventral: 1.6 mm). On day 3 after surgery, the dummy cannula was removed from the guide cannula, and then replaced by infusion cannulas (30-gauge) connected by polyethylene tubing. ADV-CMV-Tgfb1-eGFP vector (TGF-β1 vector, 1 × 10^10^ pfu/ml), ADV-CMV-eGFP control vector (control vector, 3 × 10^10^ pfu/ml), 5-aza-CdR (200 ng/μl) or vehicle was injected into the bilateral CA1 regions with 0.5 μl/side using a stepper motorized micro-syringe (Stoelting) at a rate of 0.3 μl/min. The injection needle was held in place for 3–5 min to minimize backflow.

### Data analysis/statistics

SPSS software was used for statistics analysis. The group data adhering to a Gaussian distribution were expressed as means ± s.e.m. Statistical comparison of the means between two groups of normal distribution was accomplished by the Student’s *t* test (two-tailed). For the cumulative probability, the comparison between two groups was analyzed with Kolmogorov–Smirnov test. Differences in means among multiple groups were analyzed using two-way (neonatal treatment × drug/vector) or three-way (neonatal treatment × drug/vector × time) ANOVA followed by the Bonferroni post hoc correction. The correlation analyses between continuous data with normal distribution were performed using Pearson correlation analyses. For statistical purposes, only one slice was studied per mouse in electrophysiological analysis. *P* < 0.05 was considered statistically significant.

## Results

### The enhancement of GABAergic function is responsible for the impairment of hippocampus-dependent memory in adult LPS mice

**Fig. 1:**
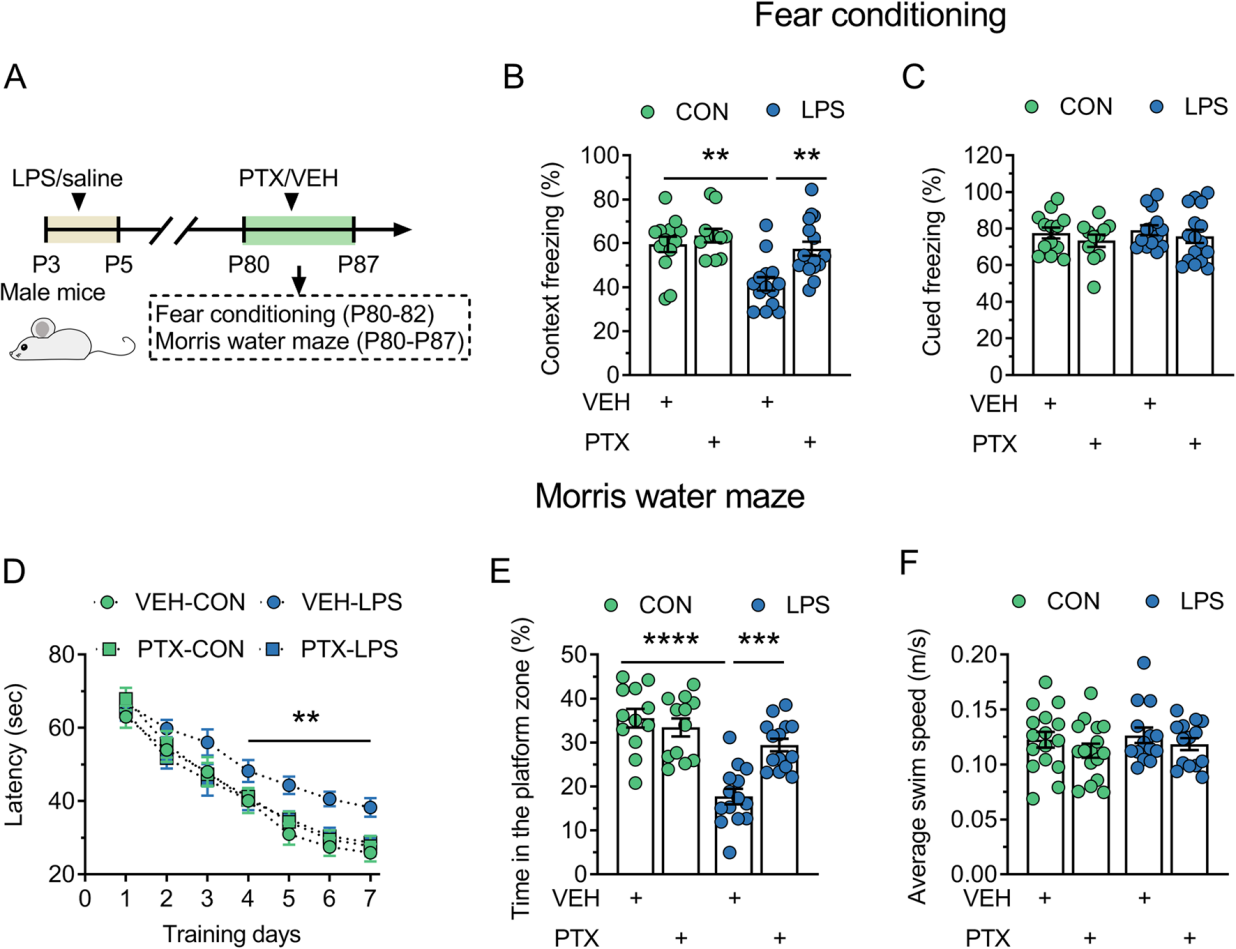
Blocking GABAergic transmission prevents the impairment of hippocampus-dependent memory in adult mice exposed to LPS during the neonatal period. CON and LPS represent control mice and mice neonatally treated with LPS (LPS mice), respectively. **A** Timeline of the experimental approach. **B** and **C** Quantification of context freezing (**B**) or cued freezing (**C**) of control mice and LPS mice treated with vehicle (VEH) or PTX in the fear conditioning. **D** Average latency (± s.e.m) to reach the hidden platform across training days of control mice and LPS mice treated with VEH or PTX in the hidden platform test of Morris water maze. **E** Quantification of time in the platform zone of control mice and LPS mice treated with VEH or PTX in the probe test of Morris water maze. **F** Quantification of the average swim speed of control mice and LPS mice treated with VEH or PTX in the probe test of Morris water maze. In **B**, **C**, **E** and **F**, circles indicate single data points and their averages (± s.e.m) are shown as columns. Statistical analysis: two-way ANOVA followed by Bonferroni in **B**, **C**, **E** and **F**, and three-way ANOVA followed by Bonferroni in D. **P < 0.01, ***P < 0.001 and ****P < 0.0001

We first investigated the role of GABAergic transmission in the effect of neonatal inflammation on adult cognition (Fig. 1A). Cognitive-related behaviors were assessed using contextual fear conditioning, cued fear conditioning and Morris water maze. The i.p. injection of PTX or vehicle was performed 45–60 min prior to training or testing every day in contextual/cued fear conditioning test as well as Morris water maze test.

#### Contextual/cued fear conditioning

Adult control and LPS mice were given a mild electric foot shock (US) with an environmental context and a tone (CS). To study contextual fear memory, which is hippocampus dependent [46], we re-exposed the mice to the same environmental context 24 h after the electric shock. To study cued fear memory in response to tone, which is hippocampus independent [46], we re-exposed the mice to the tone after the electric shock. The percentage of time that each mouse spent freezing in the same context or in response to the tone was used to assess memory. LPS mice displayed similar freezing behavior to control mice during electric shock (data not shown), suggesting the normal peripheral pain perception. However, in the contextual test conducted 24 h after the shock, LPS mice showed a decrease in freezing to the context compared to control mice (*P* = 0.002; Fig. 1B), indicating the impairment of contextual fear memory. The blockade of GABAergic transmission using PTX reversed the contextual fear memory deficits in LPS mice (*P* = 0.004) without affecting that in control mice (*P* = 0.413). The above data suggest that neonatal inflammation results in the contextual fear memory deficits via potentiating GABAergic function***.*** Notably, there were no significant differences in cued fear memory in response to the conditioned tone among control group, PTX-treated control group, LPS group and PTX-treated LPS group (neonatal treatment, F (1, 50) = 0.352, *P* = 0.556; drug, F (1, 50) = 1.337, *P* = 0.253; interaction, F (1, 50) = 0.015, *P* = 0.904; Fig. 1C). The findings indicate that neonatal inflammation causes hippocampus-dependent memory damage via intervening solely with the GABAergic transmission in the hippocampus.

#### Morris water maze test

Here, the hidden-platform water maze task and probe test were carried to further assess hippocampal-dependent memory formation. In the hidden-platform water maze task, LPS mice took more time to reach the hidden platform during training than control mice on day 4–7 (*P* = 0.001–0.008; Fig. 1D) when both groups swam at the same speed (*P* = 0.696; Fig. 1F). A probe test was carried out on day 8. In comparison with control mice, LPS mice had a significant decrease in the time spent in the platform zone (*P* < 0.0001; Fig. 1E). Without affecting the swimming speed (*P* = 0.385), PTX treatment rectified the time to the hidden-platform (*P* = 0.002–0.006) and the time in the platform zone in LPS group (*P* = 0.0001), but had no effect on those in control group (*P* = 0.079–0.362). These data again support the involvement of the potentiation of GABAergic function in hippocampal-dependent memory deficit induced by neonatal inflammation.

### The enhancement of GABAergic synaptic transmission inhibits the LTP induction in the dorsal CA1 of adult LPS mice

**Fig. 2:**
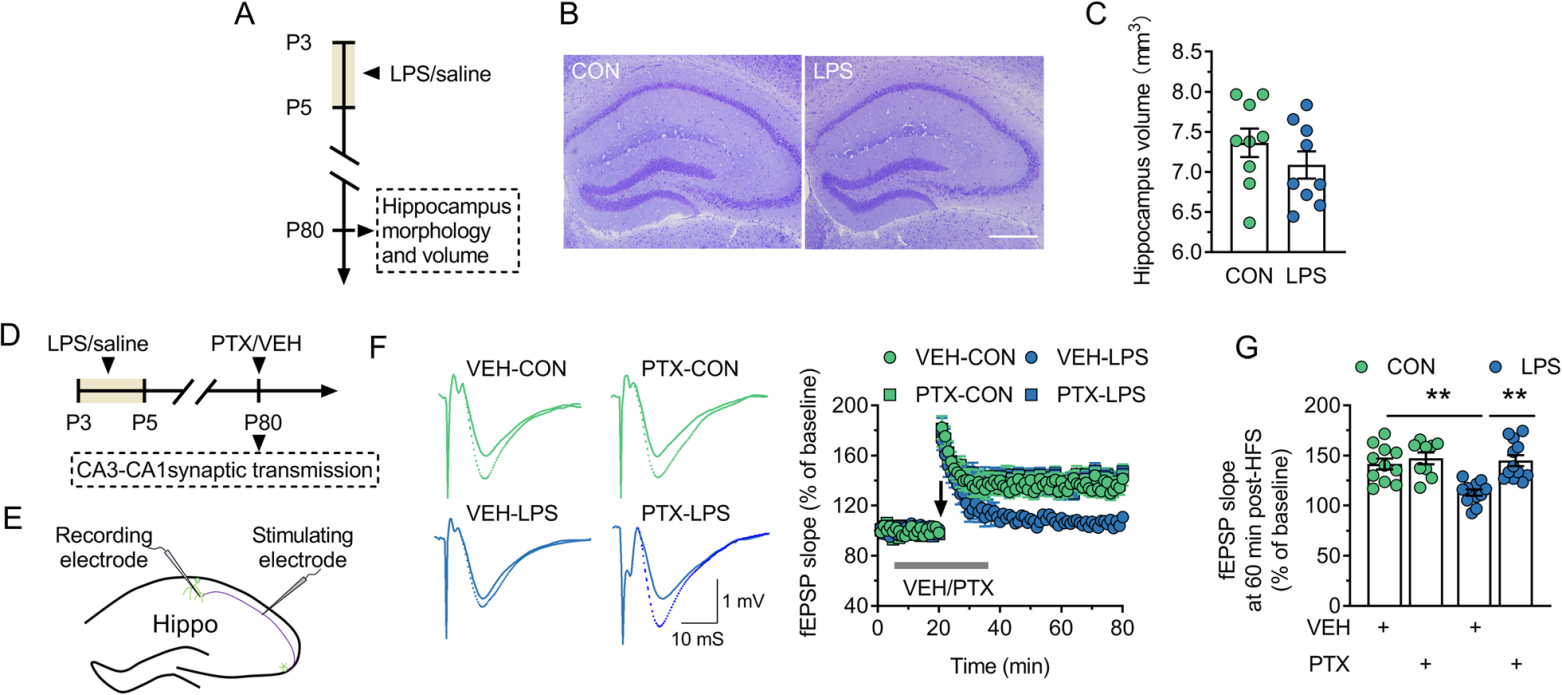
Blocking GABAergic synaptic transmission with GABA_A_R antagonist PTX (10 μM) rescues the LTP failure in dorsal CA1 of adult mice neonatally treated with LPS. CON and LPS represent control mice and mice neonatally treated with LPS (LPS mice), respectively. **A** Timeline of the experimental approach for obtaining the results in **B** and **C**. **B** and **C** The comparison of morphological structure (**B**) and volume (**C**) of the dorsal hippocampus between control and LPS mice. **B** Scale bar = 500 μm. **D** Timeline of the experimental approach for obtaining the results in **F**–**G**. **E** Sketch of the in vitro recording configuration. **F** Left, the superimposition of the original fEPSPs waves recorded in each group at 15 min (solid line) pre-HFS and 60 min post-HFS (dotted line). Right, the average time course of the change in the fEPSPs slope (± s.e.m) in hippocampal slices from control and LPS mice during bath application of either VEH or PTX. ‘↓’ indicates the time of HFS delivery. **G** The comparison of the fEPSPs slope at 60 min post-HFS in each group recorded in **F**. In **C** and **G**, circles indicate single data points and their averages (± s.e.m) are shown as columns. Statistical analysis: Student’s *t* test in **C**, two-way ANOVA followed by Bonferroni in **G**. **P < 0.01

Here, we first examined the effect of neonatal inflammation on the morphological structure or volume of the dorsal hippocampus during adulthood (Fig. 2A). It was found that neurons in all regions of the dorsal hippocampus (CA1-CA3 and DG) in LPS mice showed a regular arrangement and had a normal cellular morphology similar to that of control mice including a round cell shape and a clearly visible nucleus (Fig. 2B). Additionally, no significant difference was observed in the volume of the dorsal hippocampus between adult control and LPS mice (*P* = 0.281; Fig. 2C). LTP, which refers to the prolonged enhancement of excitatory synaptic transmission, is a widely studied cellular model of learning and memory [47]. We next investigated whether neonatal inflammation impaired the LTP in the dorsal CA1via affecting GABAergic synaptic transmission (Fig. 2D). Figure 2E shows the placement of the stimulation electrodes and the recording site in the hippocampus. As shown in Fig. 2F and G, in the slices from control mice, the slope of the fEPSPs increased to 141.1% ± 5.46% of baseline 60 min after HFS, which suggests the successful induction of LTP. In contrast, in the slices from LPS mice, the HFS protocol was not able to induce LTP (fEPSPs slope 60 min after HFS, 108.6% ± 3.63% of baseline). Pharmacological inhibition of GABAergic synaptic transmission in hippocampal slices by 30-min application of PTX (10 µM) had no effect on LTP amplitude in control slices (fEPSPs slope 60 min after HFS, 147.3% ± 5.85% of baseline) but facilitated the induction of LTP in LPS slices (fEPSPs slope 60 min after HFS, 145.0% ± 5.48% of baseline). These results suggest that neonatal inflammation inhibits the LTP induction in the dorsal CA1 of adult mice through the potentiated GABAergic synaptic transmission.

### Post-synaptic potentiation of GABAergic transmission in the dorsal CA1 is detected in adult LPS mice

**Fig. 3:**
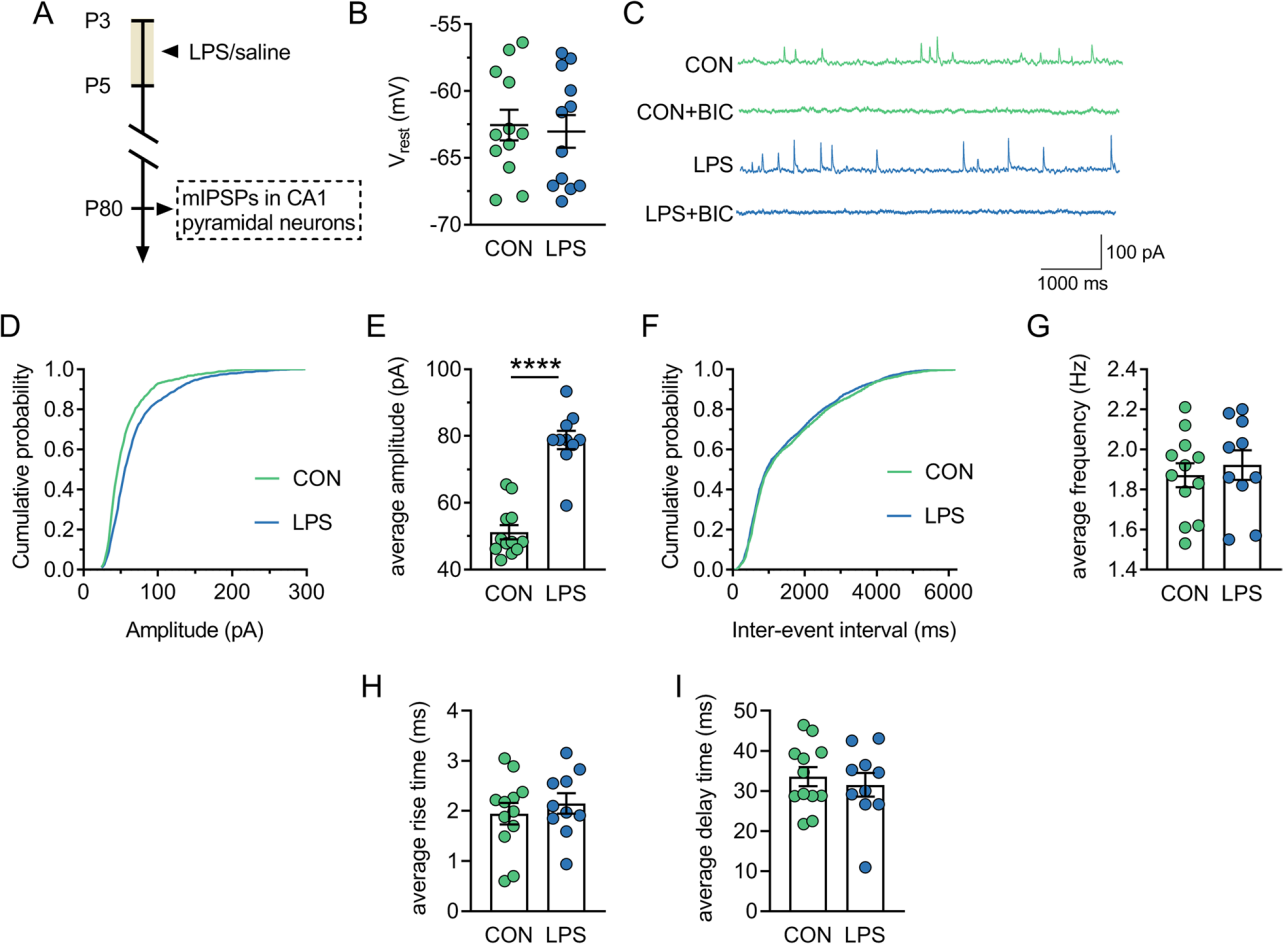
There is a postsynaptic potentiation of GABAergic transmission in dorsal CA1 of adult mice neonatally treated with LPS. CON and LPS represent control mice and mice neonatally treated with LPS (LPS mice), respectively. **A** Timeline of the experimental approach. **B** Resting potential of pyramidal neurons from control and LPS mice. **C** Representative traces of spontaneous subthreshold activity in slices from control and LPS mice before and during bath application of bicuculline (BIC). **D** Cumulative amplitude distributions of mIPSCs in pyramidal neurons from control and LPS mice. **E** Average mIPSC amplitudes in pyramidal neurons from control and LPS mice. **F** Cumulative frequency distributions of mIPSCs in pyramidal neurons from control and LPS mice. **G** Average mIPSCs frequency in pyramidal neurons from control and LPS mice. **H** Average mIPSC rise time in pyramidal neurons from control and LPS mice. **I** Average mIPSC delay time in pyramidal neurons from control and LPS mice. In **B**, **E** and **G**–**I**, circles indicate single data points and their averages (± s.e.m) are shown as columns. Statistical analysis: Student’s *t* test in **B**, **E** and **G**–**I**, and Kolmogorov–Smirnov test in **D** and **F**. ****P < 0.0001

To investigate whether presynaptic or postsynaptic mechanisms lead to the enhanced GABA transmission in adult LPS mice, we used gramicidin-perforated whole-cell recordings, a technique that preserves the endogenous intracellular Cl^−^ concentration ([Cl^−^]i) [48], to detect mIPSCs in dorsal CA1 pyramidal neurons in acute slices from adult control mice and LPS mice (Fig. 3A). First, the resting membrane potential (*V*_rest_) of control and LPS groups was about − 60 mV (Fig. 3B), showing no difference (*P* = 0.782). Spontaneous synaptic currents were then recorded at the holding potential of − 60 mV in the presence of TTX (0.4 μM), DNQX (20 M) and KN (1 mM). All spontaneous outward events were abolished by the selective GABA_A_R antagonist bicuculline (40 μM; Fig. 3C), indicating that they were mIPSCs mediated by GABA_A_R.

There was the significant rightward shift for the cumulative probability curve of mIPSCs amplitude (*P* = 0.001; Fig. 3D) and the increase of the average mIPSC amplitude (*P* < 0.0001; Fig. 3E) in LPS slices. However, Neither the position of the cumulative probability of mIPSCs frequency (*P* = 0.3; Fig. 3F) nor the average mIPSC frequency (*P* = 0.59; Fig. 3G) was altered in LPS slices. These data indicate that neonatal inflammation results in the postsynaptic potentiation of GABAergic transmission. According to the results that there was no change in the average rise time (*P* = 0.513; Fig. 3H) or the average delay time (*P* = 0.592; Fig. 3I) of mIPSCs in LPS slices, it is concluded that neonatal inflammation does not affect the average open time of GABA_A_R.

### KCC2 expression and activity are increased in the dorsal CA1 of adult LPS mice

**Fig. 4:**
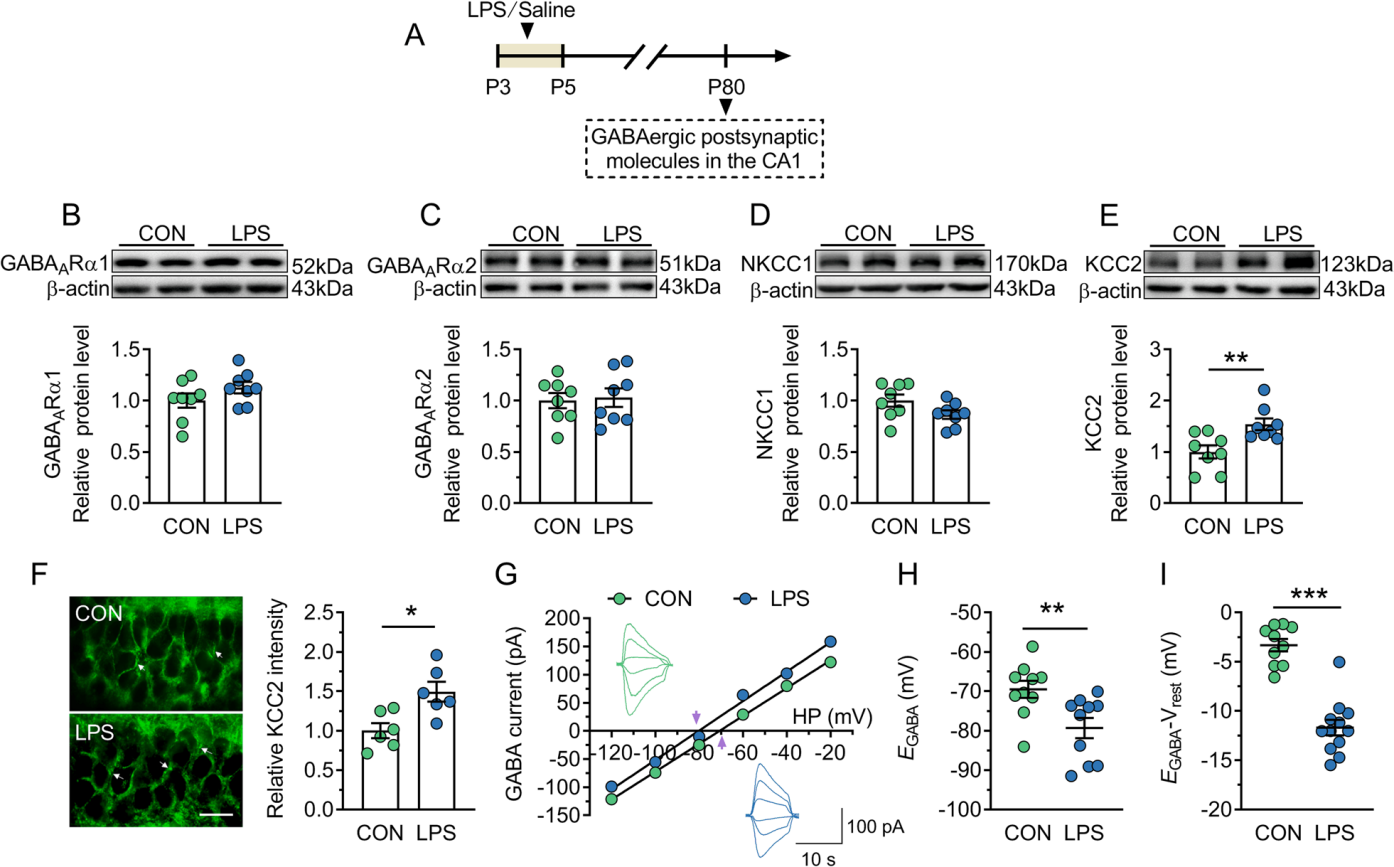
The increase in KCC2 expression and activity is found in dorsal CA1 of adult mice neonatally treated with LPS. CON and LPS represent control mice and mice neonatally treated with LPS (LPS mice), respectively. **A** Timeline of the experimental approach. **B**–**E** Top, representative immunoblots for GABA_A_Rα1 (**B**), GABA_A_Rα2 (**C**), NKCC1 (**D**) and KCC2 (**E**) in protein extracts from samples of control and LPS mice. β-actin was used as an internal standard. Bottom, quantification of GABA_A_Rα1 (**B**), GABA_A_Rα2 (**C**), NKCC1 (**D**) and KCC2 (**E**) in samples from control and LPS mice. **F** Left, representative image of immunohistostaining of KCC2. KCC2 is shown in green (white arrows). Right, the relative intensity of KCC2 in dorsal CA1 of control and LPS mice. Scale bar = 25 μm. **G** Example current–voltage curves of IPSC recorded at different holding potentials from − 120 to − 20 mV in pyramidal neurons from control and LPS mice. Insets show sample traces of *I*_GABA_ recorded in control (green) and LPS (blue) groups. *E*_GABA_ is shown at the arrow. **H** and **I** *E*_GABA_ (**H**) and driving force (**I**) measured in control and LPS groups. In **B**–**F**, **H** and **I**, circles indicate single data points, and their averages (± s.e.m.) are shown as columns in **B**–**F** or bars in **H** and **I**. Statistical analysis: Student’s *t* test. *P < 0.05, **P < 0.01 and ***P < 0.001

The effect of neonatal LPS exposure on expression profiles of GABAergic major postsynaptic signaling molecules in the dorsal CA1 was further determined in adult mice (Fig. 4A). As shown in Fig. 4B–D, there was no significant difference in protein expression of GABA_A_Rα1, GABA_A_Rα2, and NKCC1 between control and LPS mice (*P* = 0.082–0.81). However, the protein level of KCC2 was significantly increased in LPS mice than that in control mice (*P* = 0.007; Fig. 4E). Furthermore, KCC2 labeling was stronger at the membrane of pyramidal cells in LPS mice (*P* = 0.011; Fig. 4F). KCC2 in neurons that is largely responsible for setting the transmembrane chloride gradient [49]. Because GABA_A_R is coupled with membrane Cl^−^ channels, proper maintenance of the transmembrane Cl^−^ gradient is critical for the polarity (hyperpolarizing vs. depolarizing) and efficacy of GABAergic function. To address whether the altered protein level of KCC2 in LPS mice disrupts the polarity of GABA, we recorded *E*_GABA_ from the CA1 pyramidal neurons in dorsal hippocampal slices using gramicidin-perforated whole-cell patch-clamp recordings. It was found that *E*_GABA_ from LPS mice was significantly hyperpolarized (− 79.31 ± 2.56 mV) compared with control mice (− 69.43 ± 2.17 mV; *P* = 0.009; Fig. 4G and H). As a result, the driving force of inward Cl^−^ currents (*E*_GABA_ − *V*_rest_) was potentiated in LPS mice (*P* < 0.001; Fig. 4I). These results suggest that neonatal inflammation hyperpolarizes *E*_GABA_ through KCC2 overexpression, which further leads to enhanced GABA-evoked postsynaptic inhibition in the dorsal CA1 of adult hippocampus.

### The excess expression of KCC2 is related with the TGF-β1 downregulation in adult LPS mice

**Fig. 5:**
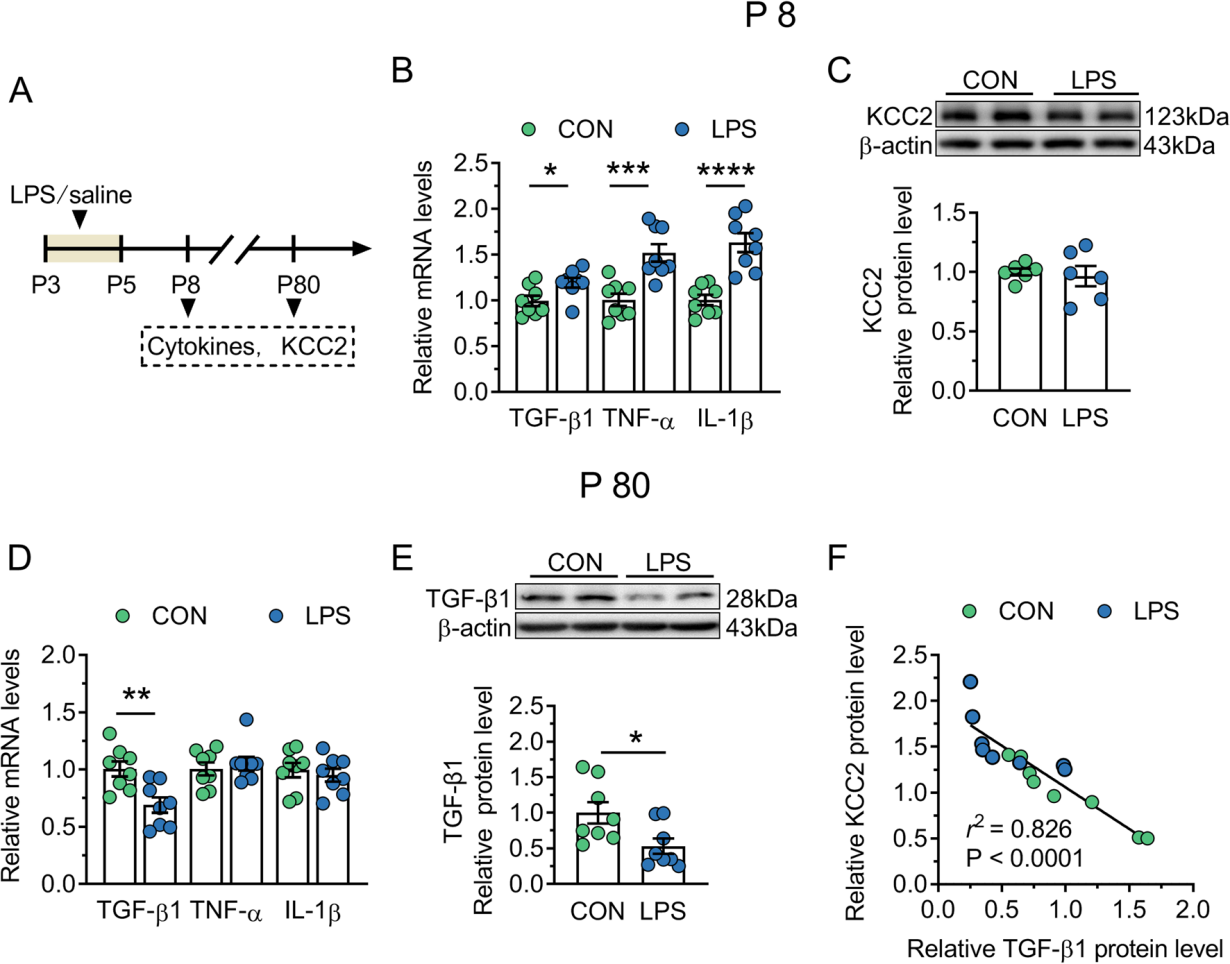
KCC2 overexpression is associated with TGF-β1 decrease in the dorsal CA1 region of adult mice neonatally treated with LPS. CON and LPS represent control mice and mice neonatally treated with LPS (LPS mice), respectively. **A** Timeline of the experimental approach. **B** and **D** Quantification of mRNA expressions of inflammatory factors in samples from P8 (**B**) or P80 (**D**) control and LPS mice. **C** Top, representative immunoblots for KCC2 in protein extracts from samples of P8 control and LPS mice. β-actin was used as an internal standard. Bottom, quantification of KCC2 in samples from P8 control and LPS mice. **E** Top, representative immunoblots for TGF-β1 in protein extracts from samples of P80 control and LPS mice. β-actin was used as an internal standard. Bottom, quantification of TGF-β1 in samples from P80 control and LPS mice. **F** Pearson analysis of the correlation between KCC2 and TGF-β1 protein in samples from P80 control and LPS mice. In **B**–**F**, circles indicate single data points. In **B**–**E**, columns represent the averages (± s.e.m.) of the data. Statistical analysis: Student’s *t* test in **B**–**E**, and Pearson analysis in **F**. *P < 0.05, **P < 0.01, ***P < 0.001 and ****P < 0.0001

Based the reports that early LPS exposure causes changes in pro- and anti-inflammatory cytokines in the brain [39], we examined the levels of IL-1β, TNF-α and TGF-β1 in the dorsal CA1 of control and LPS mice at P8 and P80 and analyzed the correlation between cytokines and KCC2 (Fig. 5A). At P8, LPS mice showed the increased expressions of IL-1β, TNF-α and TGF-β1 at mRNA (*P* = 0.0001–0.024; Fig. 5B) or protein level (*P* = 0.003–0.023; Additional file 1: Fig. S1A–C) compared with control mice. However, the KCC2 protein expression showed no difference between two groups (*P* = 0.71; Fig. 5C). At P80, TGF-β1 mRNA or protein expression was significantly lower in LPS group (mRNA, *P* = 0.005; protein, *P* = 0.024; Fig. 5D and E). However, there was no difference in the expression of IL-1β or TNF-α at mRNA (*P* = 0.493–0.615) and protein level (*P* = 0.06–0.241; Additional file 1: Fig. S1D–E) between control and LPS groups. We further analyzed the relation between TGF-β1 protein and KCC2 protein and found that the protein expression of KCC2 was negatively correlated with TGF-β1 protein (*r*^2^ = 0.826, *P* < 0.0001; Fig. 5F). These results suggest that the KCC2 overexpression in LPS mice during adulthood is possibly associated with the lack of TGF-β1 at this time.

### TGF-β1 downregulation contributes to the increase of KCC2 expression and function in adult LPS mice

**Fig. 6:**
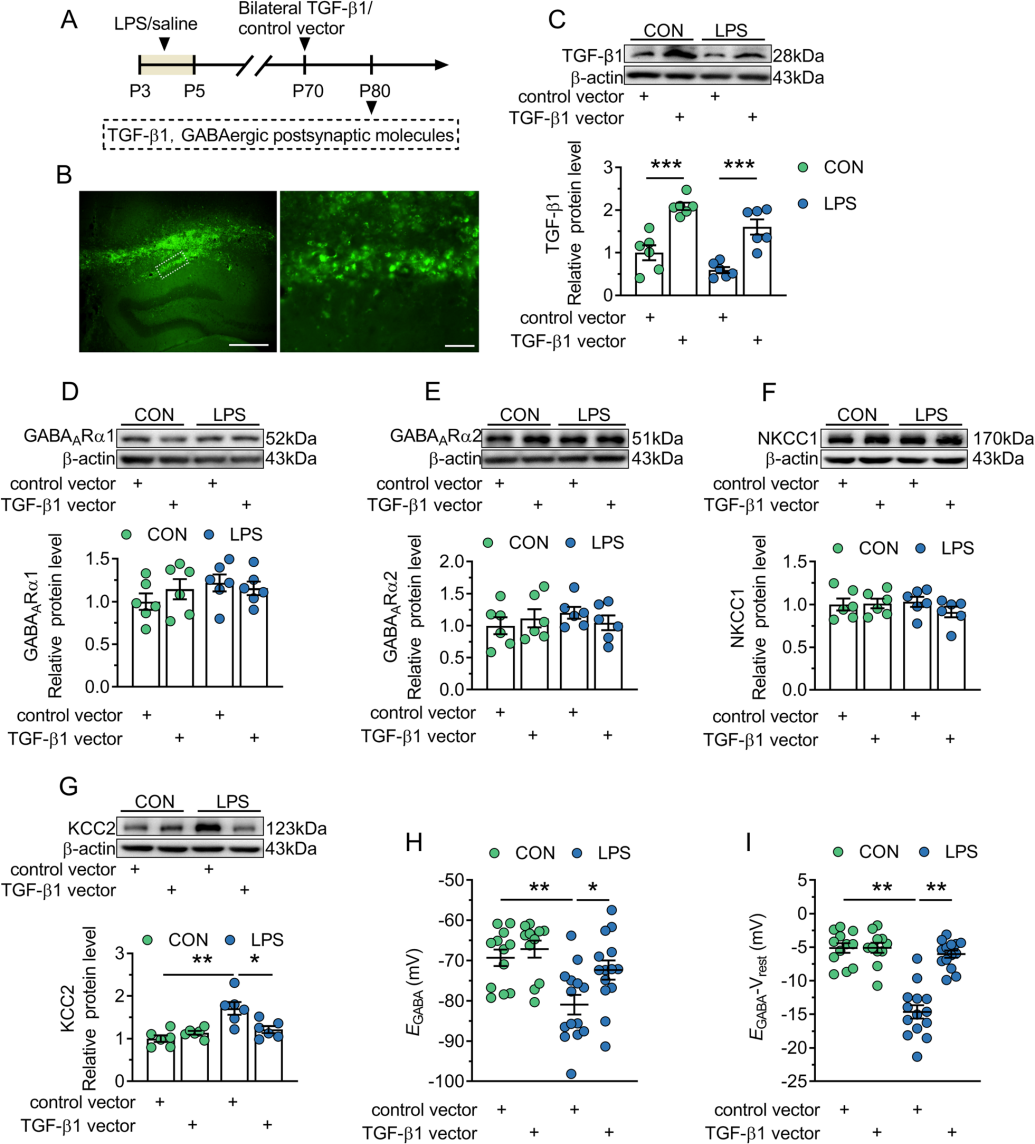
TGF-β1 vector treatment rescues KCC2 expression and function in the dorsal CA1 of adult mice neonatally treated with LPS. CON and LPS represent control mice and mice neonatally treated with LPS (LPS mice), respectively. **A** Timeline of the experimental approach. **B** Left, abundant expression of eGFP (green) in the dorsal CA1 region. Scale bar = 500 μm. Right, expression of eGFP in cells in the CA1 region with the white dashed box in the left panel. Scale bar = 50 μm. **C**–**G**: Top, representative immunoblots for TGF-β1 (**C**), GABA_A_Rα1 (**D**), GABA_A_Rα2 (**E**), NKCC1 (**F**) and KCC2 (**G**) in protein extracts from samples of control and LPS mice treated with control vector or TGF-β1 vector. β-actin was used as an internal standard. Bottom, quantification of TGF-β1 (**C**), GABA_A_Rα1 (**D**), GABA_A_Rα2 (**E**), NKCC1 (**F**) and KCC2 (**G**) in samples from control and LPS mice treated with control vector or TGF-β1 vector. **H** and **I** *E*_GABA_ (**H**) and driving force (**I**) measured in each experimental group. In **C**–**I**, circles indicate single data points, and their averages (± s.e.m.) are shown as columns in **C**–**G** or bars in **H** and **I**. Statistical analysis: two-way ANOVA followed by Bonferroni. *P < 0.05, **P < 0.01 and ***P < 0.001

Here, we speculated that local, chronic TGF-β1 upregulation in the adult dorsal CA1 would at least partially restore KCC2 expression. TGF-β1 vector or control vector was bilaterally injected into the dorsal CA1 of adult control and LPS mice 10 days prior to the detection (Fig. 6A). As shown in Fig. 6B, a substantial amount of eGFP (green) was detected in the dorsal CA1 region, which confirmed the successful expression of control vector or TGF-β1 vector. Western blot analysis in Fig. 6C further showed that TGF-β1 protein greatly enhanced by TGF-β1 vector in control mice and LPS mice when compared with control vector-treated control mice (*P* = 0.0002) and LPS mice (*P* = 0.0003), respectively. Although there was no effect of TGF-β1 vector on protein levels of GABA_A_Rα1, GABA_A_Rα2 and NKCC1 in control and LPS mice (*P* = 0.311–0.775; Fig. 6D–F). TGF-β1 vector reduced KCC2 protein in LPS mice to the control level (*P* = 0.0158; Fig. 6G) without affecting that in control mice (*P* = 0.151). The altered *E*_GABA_ or the driving force of inward Cl^−^ currents was also rectified by TGF-β1 vector in LPS mice (*E*_GABA_, *P* = 0.184; *E*_GABA_ − V_rest_, *P* = 0.0021; Fig. 6H and I). The results suggest that TGF-β1 downregulation is the cause for the increased expression and function of KCC2 in adult LPS mice.

### TGF-β1 downregulation contributes to the impairment of synaptic plasticity and hippocampus-dependent memory of adult LPS mice

**Fig. 7:**
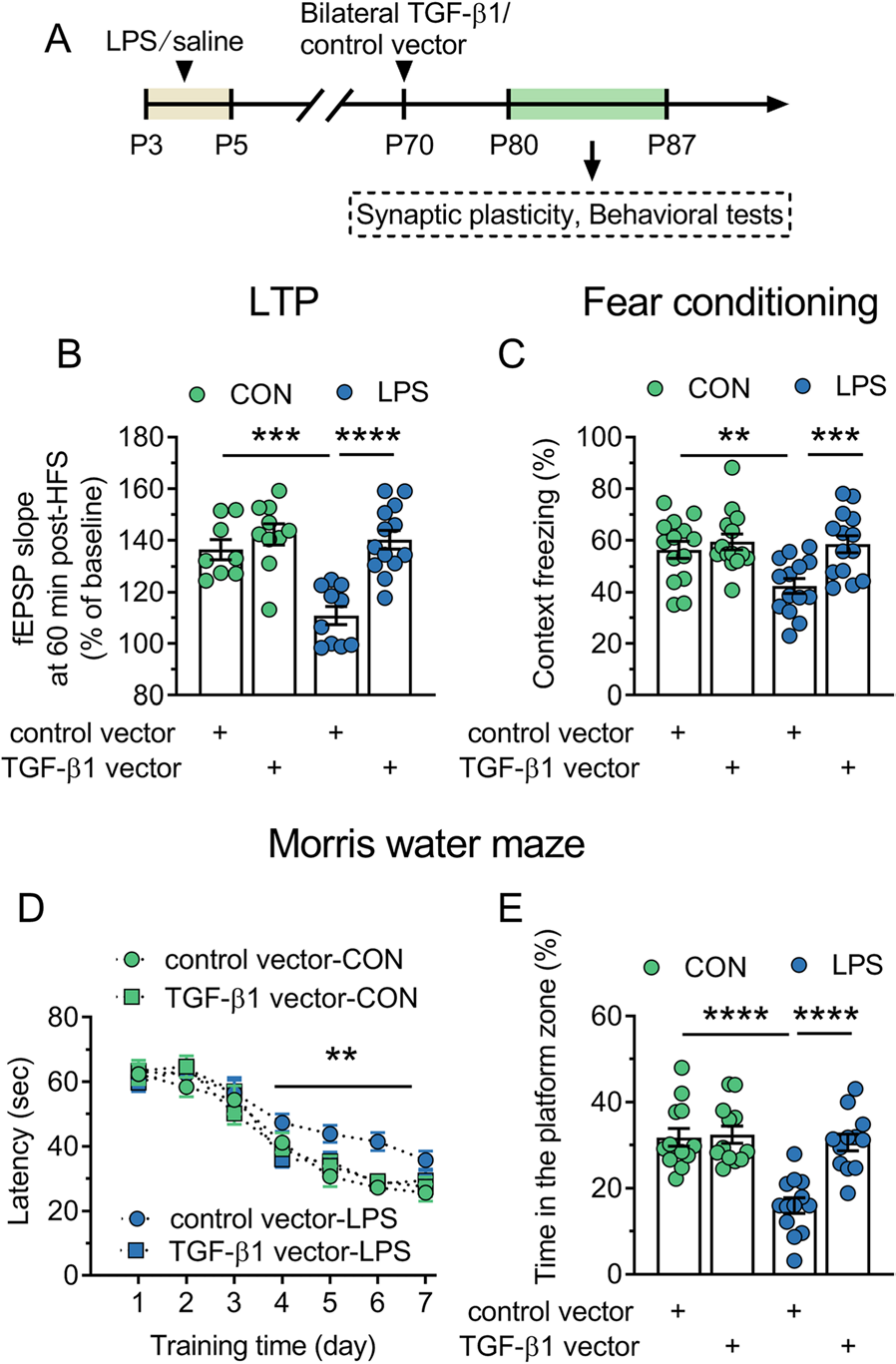
TGF-β1 vector treatment restores the synaptic plasticity in the dorsal CA1 area and hippocampus-dependent memory in adult mice neonatally treated with LPS. CON and LPS represent control mice and mice neonatally treated with LPS (LPS mice), respectively. **A** Timeline of the experimental approach. **B** Quantification of synaptic plasticity at CA3–CA1 pathway in control and LPS mice treated with control vector or TGF-β1 vector. **C** Quantification of context freezing of control and LPS mice treated with control vector or TGF-β1 vector in the fear conditioning. **D** Average latency (± s.e.m) to reach the hidden platform across training days of control mice and LPS mice treated with control vector or TGF-β1 vector in the hidden platform test of Morris water maze. **E** Quantification of time in the platform zone of control and LPS mice treated with control vector or TGF-β1 vector in the probe test of Morris water maze. In **B**, **C**, and **E**, circles indicate single data points and their averages (± s.e.m) are shown as columns. Statistical analysis: two-way ANOVA followed by Bonferroni in **B**, **C** and **E**, and three-way ANOVA followed by Bonferroni in **D**. **P < 0.01, ***P < 0.001 and ****P < 0.0001

As KCC2 overexpression resulted in the enhancement of GABA inhibition which is associated with impaired hippocampal synaptic plasticity and cognitive function, we further investigated the possible role of TGF-β1 downregulation in hippocampal LTP impairment and memory deficits by the administration of TGF-β1 vector or control vector (Fig. 7A). We found that TGF-β1 vector completely rescued the LTP induction in LPS mice and restored it to the level observed in control mice (*P* < 0.0001; Fig. 7B). Conversely, we found no effect of TGF-β1 vector on LTP in control mice (*P* = 0.322). Additionally, TGF-β1 vector rectified a decrease in freezing to the context of LPS mice (*P* = 0.0003; Fig. 7C), indicating a recovery of hippocampus-dependent memory performance. Consistently, LPS mice with TGF-β1 vector administration performed as well as control mice in Morris water maze. In LPS mice, TGF-β1 vector rescued the prolonged escape latency to the hidden-platform during the training (*P* = 0.002–0.009; Fig. 7D) and the decreased time in the platform zone during the probe trial (*P* < 0.0001; Fig. 7E). Combined with the results in Fig. 6, it is believed that TGF-β1 downregulation increases the expression and function of KCC2 in the dorsal CA1 area, which impairs synaptic plasticity and -related memory of adult LPS mice.

### Hypermethylation at TGFb1 promoter is the underlying mechanism of downregulation of TGF-β1 expression in adult LPS mice

**Fig. 8:**
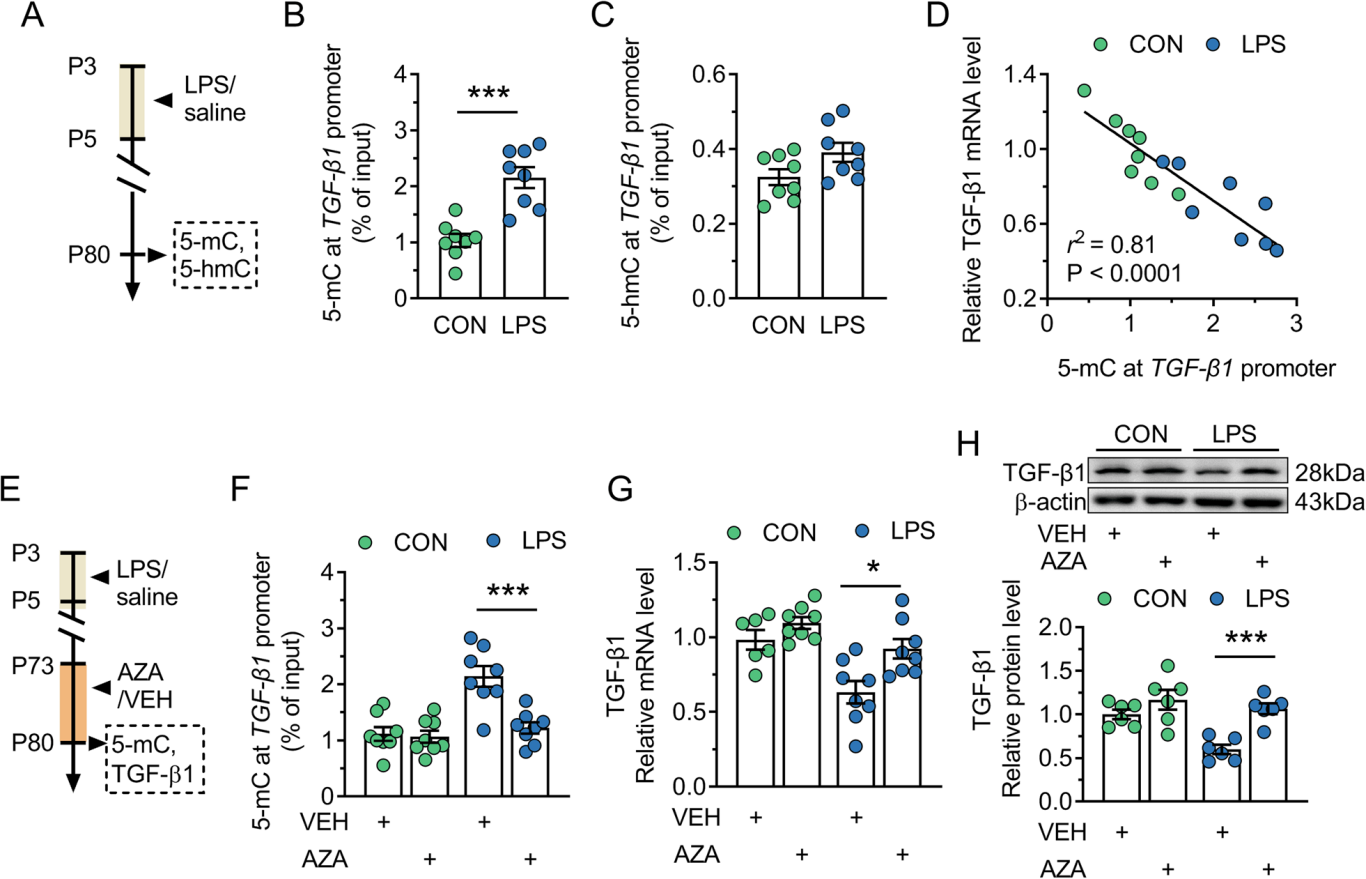
Hypermethylation at *TGFb1* promoter is responsible for the decreased TGF-β1 expression in adult mice neonatally treated with LPS. CON and LPS represent control mice and mice neonatally treated with LPS (LPS mice), respectively. **A** Timeline of the experimental approach for obtaining the results in **B**–**D**. **B** and **C** Quantification of 5-mC (**B**) and 5-hmC (**C**) at *TGFb1* promoter from samples of control and LPS mice. **D** Pearson analysis of the correlation between 5-mC at *TGFb1* promoter and the corresponding transcripts in samples from adult control and LPS mice. **E** Timeline of the experimental approach for obtaining the results in **F**–**H**. **F** Quantification of 5-mC at *TGFb1* promoter from samples of adult control and LPS mice treated with VEH or 5-aza-CdR (AZA). **G**: Quantification of TGF-β1 mRNA expression from samples of adult control and LPS mice treated with VEH or AZA. **H** Top, representative immunoblots for TGF-β1 in protein extracts from samples of control and LPS mice treated with VEH or AZA. β-actin was used as an internal standard. Bottom, quantification of TGF-β1 protein in samples from control and LPS mice treated with VEH or AZA. In **B**–**D** and **F**–**H**, circles indicate single data points. In **B**, **C** and **F**–**H**, columns represent the averages (± s.e.m.) of the data. Statistical analysis: Student’s *t* test in **B** and **C**, Pearson analysis in **D**, and two-way ANOVA followed by Bonferroni in **F**–**H**. *P < 0.05, and ***P < 0.001

A latest study by Pierre et al. [50] has pointed out that postnatal inflammatory exposure caused long-lasting cerebral methylation changes in genes relevant to inflammatory response. Based on the fact that there is the enrichment of CpG islands in the promoter region of *TGFb1* gene, it was further determined whether TGF-β1 decrease was caused by abnormal methylation of *TGFb1* promoter region (Fig. 8A). 5-mC and 5-hmC are the two most important DNA epigenetic modifications. Here, we found that there was a significant enrichment of 5-mC at *TGFb1* promoter in LPS mice when compared with control mice (*P* = 0.0002; Fig. 8B). However, 5-hmC level at *TGFb1* promoter was not changed in LPS mice (*P* = 0.064; Fig. 8C). Notably, the enrichment of 5-mC at *TGFb1* promoter was negatively correlated with the level of corresponding TGF-β1 transcript (*r*^*2*^ = 0.81, *P* < 0.0001; Fig. 8D). This result suggests promoter methylation may be an epigenetic mechanism leading to reduced TGF-β1 in LPS mice, and this possibility was therefore examined via detecting the effect of intra-CA1 injection of methylation inhibitor 5-aza-CdR on 5-mC at *TGFb1* promoter and TGF-β1 expression (Fig. 8E). The results showed that repeated 5-aza-CdR treatment significantly rectified not only the hypermethylation of *TGFb1* promoter (*P* = 0.0007; Fig. 8F), but also the decreased TGF-β1 mRNA (*P* = 0.011; Fig. 8G) and protein expression (*P* = 0.0002; Fig. 8H) of LPS mice. The above data strongly suggest that the pathological decline of TGF-β1 expression in adult LPS mice is caused by hypermethylation at *TGFb1* promoter.

## Discussion

Using a combination of behavioral studies, electrophysiological recordings, and biochemical methods together with pharmacological tools, the present study provides the first evidence that early inflammation induced by neonatal LPS exposure promotes KCC2 expression and function in principal neurons of dorsal CA1 area through hypermethylation-induced reduction of TGF-β1, which thereby potentiates GABAergic synaptic inhibition leading to hippocampus-dependent memory deficits in adulthood.

The brain development during early life is an intricate and subtle process and greatly sensitive to environmental insults. Adverse early-life agents are at increased risk for the development of adult neuropsychiatric diseases [51, 52]. Inflammation is one of the most common adverse factors and LPS is a potent inducer of inflammation widely used for preparing models of infection in prenatal, neonatal, juvenile and adult periods [53–55]. In the present study, neonatal inflammation impaired hippocampus-dependent memory (contextual and spatial memory) but not hippocampus-independent memory (cued fear memory) in adult mice. This may be related to the fact that the hippocampus is highly sensitive to inflammation due to its large blood flow and abundant inflammatory factor receptors [56]. Hippocampus CA1 area is reported to be involved in the consolidation process of contextual memory [57] and acquisition and consolidation of spatial memory [58]. It is inferred that neonatal inflammation impairs contextual or spatial memory at acquisition and consolidation stages.

The treatment with the GABA_A_R antagonist PTX (10 µM) successfully rescued the failure of LTP induction in the dorsal CA1 region and the behavioral deficits associated with hippocampal-dependent memory in adult LPS mice, without affecting the corresponding synaptic plasticity and behaviors in control mice. This result suggests that the enhancement of GABAergic synaptic transmission is responsible for the damaging effects of neonatal inflammation on hippocampus-dependent memory. Using patch-clamp techniques, we found that there was a marked increase in the amplitude but not frequency of GABAergic mIPSCs in adult LPS mice, which suggests that enhanced GABAergic synaptic transmission in adult LPS mice is caused by changes in postsynaptic function. Based on the results that there was no difference in the rise time and delay time of average mIPSCs between control and LPS mice, it can be concluded that neonatal inflammation does not affect the average open time of GABA_A_R which mediates mIPSCs.

It should be noted that there are some limitations about the present study on the influence of neonatal inflammation on GABAergic synaptic transmission. First, mIPSCs can be divided into two groups, i.e., slow and fast rising mIPSCs, which may be mediated by different synaptic GABA_A_R [59]. Since no distinction was made between fast mIPSCs and slow mIPSCs, it could not be determined whether neonatal inflammation had an effect on the kinetics of GABA_A_R that mediates fast mIPSCs or slow mIPSCs. Second, synaptic GABA_A_R mediates mIPSCs which belongs to phasic inhibition while extrasynaptic GABA_A_R mediates tonic inhibition [60]. In the CA1 subfield of the hippocampus, the generation of tonic GABA current depends on the α5-subunit-containing subtype of GABA_A_R (α5GABA_A_R) [61]. There is some evidence indicating that tonic inhibition generated by α5GABA_A_R plays a role in the regulation of memory. For example, drugs that increase α5GABA_A_R activity cause profound memory blockade [62, 63]. Since tonic GABA current was not examined, the exact role of tonic inhibition in neonatal inflammation-induced cognitive impairment could not be determined.

In most mature neurons, GABA_A_R opens a chloride channel and chloride anions enter the cell to hyperpolarize the cell membrane. This influx of chloride is supported by [Cl^−^]i gradient between the inside and the outside part of the membrane which is maintained by the activity of chloride cotransporters such as NKCC1 and KCC2, a chloride importer and a chloride exporter, respectively. In mature tissue of control animals, KCC2 is highly expressed as compared with NKCC1, resulting in a net efflux of chloride to maintain a low internal [Cl^−^]i. Interestingly, during development, GABA_A_R can exert excitatory effects as a consequence of elevated [Cl^−^]i in immature neurons, which express less KCC2 [64]. During maturation, the increasing expression of KCC2 is a key factor in the switch of GABA_A_R to inhibitory function [49, 65]. Among several postsynaptic proteins of GABAergic synapse, only the expression KCC2 was increased in adult LPS mice in the present study. Corresponding, adult LPS mice was found to show the hyperpolarization of *E*_GABA_ in the principal neurons. Based these results, it is believed that the potentiation of GABAergic synaptic inhibition in adult LPS mice may be related with the increase in the KCC2 expression and function. Impaired synaptic inhibition due to decreased KCC2 function has been documented in Huntington’s disease [66], epilepsy [67, 68], neuropathic pain [69] and spasticity following spinal cord injury [70]. A report by Silvestre de Ferron et al. [71] has pointed out that an upregulation of KCC2 expression after perinatal ethanol exposure leads to the deficits of synaptic plasticity in the hippocampus via increased GABAergic inhibition. Based on these results, increased expression and activity of KCC2 provide the explanation for the enhanced GABAergic synaptic inhibition and memory deficits of adult LPS mice. A recent study has found that there are both pre- and post-synaptic potentiations of GABAergic transmission in the prefrontal cortex accompanied with increased expressions of multiple GABAergic synapse-associated proteins 2 h after LPS challenge [72]. The study combined with our research suggests that there is difference in acute and long-term effects of inflammation on GABAergic synaptic transmission, which may be related to the involvement of different molecular mechanisms.

Systemic LPS administration can result in the inflammatory response of brain-resident immune cells which are mainly microglia and astrocytes and a myriad release of pro- or anti-inflammatory cytokines [73]. Although the activation of microglia or astrocytes was not examined, we found an acute increase in mRNA expressions of IL-1β, TGF-α and TGF-β1 in the dorsal CA1 region after neonatal LPS treatment, which still suggests that LPS triggers local inflammation. Based on the results that LPS mice showed similar levels of IL-1β and TGF-α as control mice, it is clarified that neonatal inflammation does not persist into adulthood. Additionally, our important finding here is that neonatal inflammation leads to a long-term reduction in TGF-β1 expression. The early development period is crucial for establishing and maintaining epigenetic marks [74]. Epigenetic mechanisms are consequently regarded as the most plausible targets through which adverse factors could exert their persistent effects [75]. Based on the findings of persistent methylation changes in the brain after early life inflammation [50] and the fact that there are enriched CpG islands at *TGFb1* promoter, it is preliminarily confirmed by the present study that hypermethylation at *TGFb1* promoter is the critical link between neonatal inflammation and TGF-β1 decrease in adulthood. DNA methyltransferases (DNMTs) and ten-eleven translocation hydroxylases (TETs) are important components of the DNA-methylation/demethylation dynamic regulating the expression of key molecules involved in brain function [76]. In the follow-up study, we will focus on investigating the effect of neonatal inflammation on DNMTs and TETs and the potential mechanisms.

Our study showed KCC2 protein was negatively correlated with TGF-β1 protein during adulthood and the local overexpression of TGF-β1 induced a decrease of KCC2 protein in adult LPS mice up to the level in control mice. These data clearly indicate that the persistent reduction in TGF-β1 caused by neonatal inflammation accounts for the increased expression of KCC2 in adulthood. It is therefore speculated that the possible acute neurological effects of neonatal inflammation have been recovered later in life. The mechanisms underlying TGF-β1 affecting KCC2 expression are still not fully understood. Rivera et al. shows that scavenging of endogenous BDNF, or inhibition of signaling downstream from TrkB receptors, effectively prevents activity-dependent reduction of KCC2 expression [77]. Other studies report that the decrease of BDNF release increases KCC2 expression and the inhibitory strength of GABA following axonal injury [70, 78, 79]. These findings suggest the activation of BDNF/TrkB signaling negatively regulates in KCC2 expression in mature neurons. A recent study points that TGF-β1 enhances the expressions of BDNF and TrkB in cerebral cortex neurons and promotes microglial secretion of BDNF [80]. Accordingly, we speculate that the decline of BDNF/TrkB pathway may be involved in the increased KCC2 expression and function induced by TGF-β1 deficit in adult LPS mice. Combined with the results that TGF-β1 overexpression recovered hippocampal LTP and -related memory in adult LPS mice, it is further believed that a reduction of TGF-β1 promotes KCC2 expression in the dorsal CA1 as one of the underlying mechanisms of neonatal inflammation resulting in hippocampal memory deficit. Additionally, a study on a mouse model of AD found that TGF-β1 improved the expressions of synaptic plasticity-related proteins including Arc, NR2B and PSD-95, as well as spatial memory through activating PI3K/Akt pathway [81], and several other studies proved that TGF-β1 increased CREB phosphorylation which is involved in various forms of synaptic plasticity and memory [82, 83]. Based on these facts, we cannot exclude that TGF-β1 deficiency mediates the damaging effects of neonatal inflammation on hippocampal memory through other ways such as decreased PI3K/Akt activity or CREB phosphorylation.

In conclusion, based on the well-documented association between early-life exposure to inflammation and cognitive impairment in adulthood, the current work depicts that hypermethylation-induced deficiency of TGF-β1 further leads to overexpression of KCC2 in the CA1 region of the dorsal hippocampus, which is the important mechanism underlying the adverse effect of neonatal inflammation on hippocampus-dependent memory. The present study reveals that TGF-β1 or KCC2 may be potential therapeutic targets in memory deficit induced by neonatal inflammation through recovering the function of hippocampal GABAergic synapse.

## Supplementary Information

**Additional file 1: Figure S1.** Neonatal LPS exposure LPS induces an acute but not persistent increase in protein expression of inflammatory factors. CON and LPS represent control mice and mice neonatally treated with LPS (LPS mice), respectively. **A**–**C** Top, representative immunoblots for IL-1β (A), TNF-α (B) and TGF-β1 (C) in protein extracts from samples of P8 control and LPS mice. β-actin was used as an internal standard. Bottom, quantification of IL-1β (A), TNF-α (B) and TGF-β1 (C) in samples from P8 control and LPS mice. **D** and **E** Top, representative immunoblots for IL-1β (D), TNF-α (E) in protein extracts from samples of P80 control and LPS mice. β-actin was used as an internal standard. Bottom, quantification of IL-1β (D), TNF-α (E) in samples from P80 control and LPS mice. Circles indicate single data points and their averages (± s.e.m) are shown as columns. Statistical analysis: Student’s *t* test. *P < 0.05 and **P < 0.01.

## Data Availability

The datasets analyzed during the current study are available from the corresponding author on reasonable request.

## Abbreviations

5-aza-CdR: 5-Aza-deoxycytidine
ACSF: Artificial cerebrospinal fluid
ADV: Adenoviral
[Cl^−^]i: Intracellular Cl^−^ concentration
CS: Conditioned stimulus
DNMTs: DNA methyltransferases
*E*_GABA_: Reversal potential for *I*_GABA_
fEPSPs: Field excitatory postsynaptic potentials
GABA: γ-Aminobutyric acid
GABA_A_R: GABA type A receptor
HFS: High-frequency stimulation
KCC2: K^+^–Cl^−^ cotransporter 2
*I*_GABA_: GABA-activated current
IL-1β: Interleukin-1beta
LPS: Lipopolysaccharide
LTP: Long-term potentiation
NKCC1: Na^+^–K^+^–Cl^−^ cotransporter 1
mIPSCs: Miniature inhibitory postsynaptic currents
P: Postnatal day
PPF: Paired-pulse facilitation
PTX: Picrotoxin
TETs: Ten-eleven translocation hydroxylases
TGF-β1: Transforming growth factor-beta 1
TNF-α: Tumor necrosis factor alpha
US: Unconditioned stimulus
*V*_rest_: Resting membrane potential
