# Supplementary figures and images for: Neonatal inflammation increases hippocampal KCC2 expression through methylation-mediated TGF-β1 downregulation leading to impaired hippocampal cognitive function and synaptic plasticity in adult mice
Source: J Neuroinflammation. 2023 Jan 23;20:15. doi: 10.1186/s12974-023-02697-x (PMC9872321; doi:10.1186/s12974-023-02697-x)

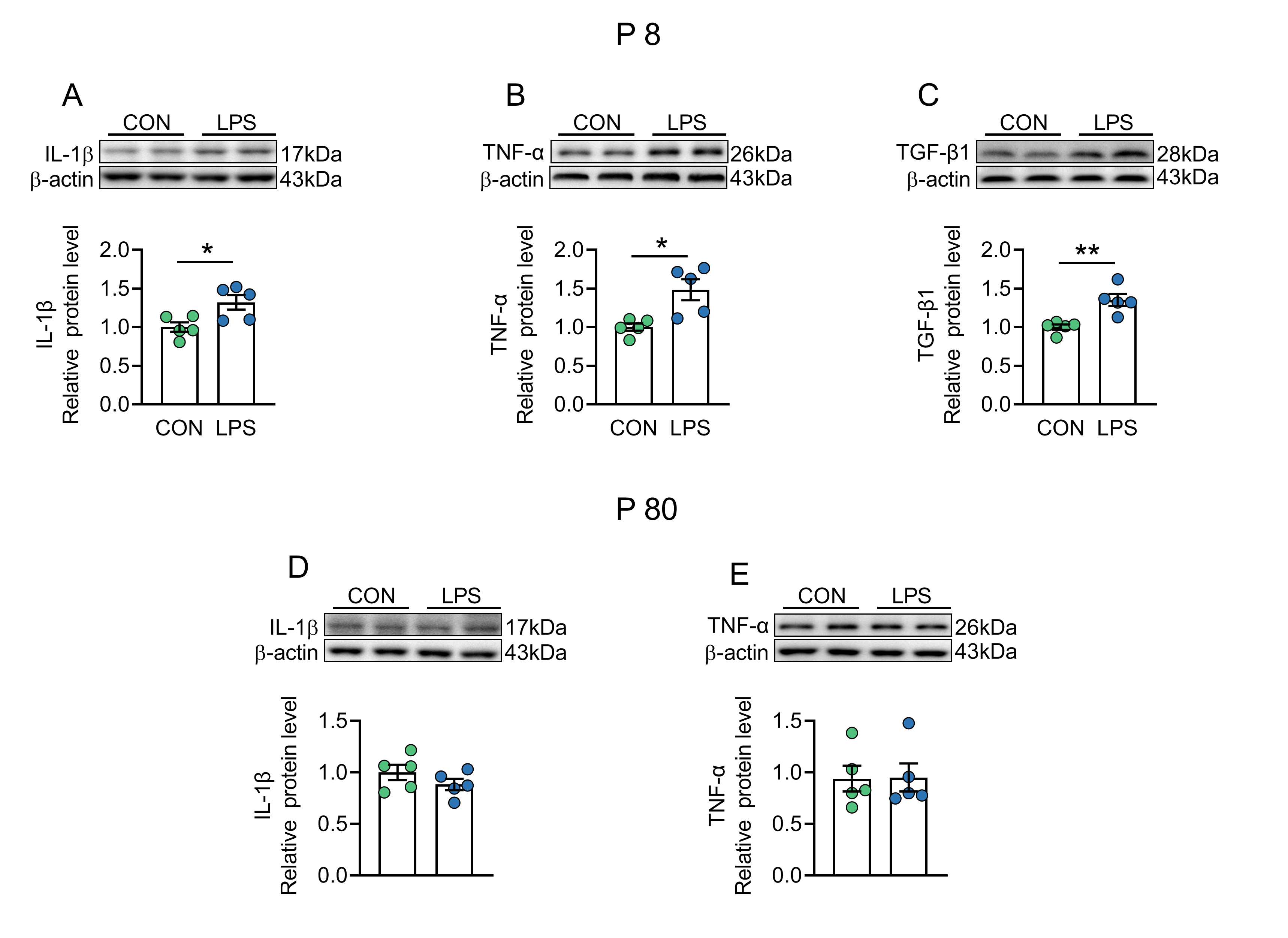

Supplement: Supplementary file 1 — Additional file 1: Figure S1. Neonatal LPS exposure LPS induces an acute but not persistent increase in protein expression of inflammatory factors. CON and LPS represent control mice and mice neonatally treated with LPS (LPS mice), respectively. A–C Top, representative immunoblots for IL-1β (A), TNF-α (B) and TGF-β1 (C) in protein extracts from samples of P8 control and LPS mice. β-actin was used as an internal standard. Bottom, quantification of IL-1β (A), TNF-α (B) and TGF-β1 (C) in samples from P8 control and LPS mice. D and E Top, representative immunoblots for IL-1β (D), TNF-α (E) in protein extracts from samples of P80 control and LPS mice. β-actin was used as an internal standard. Bottom, quantification of IL-1β (D), TNF-α (E) in samples from P80 control and LPS mice. Circles indicate single data points and their averages (± s.e.m) are shown as columns. Statistical analysis: Student’s t test. *P < 0.05 and **P < 0.01. [file 12974_2023_2697_MOESM1_ESM.tif]
